# Associations between air pollution and relative leukocyte telomere length among northern Swedish adults based on findings from the Betula study

**DOI:** 10.1038/s41598-025-19469-7

**Published:** 2025-09-23

**Authors:** Wasif Raza, Sara Pudas, Katja M. Kanninen, Erin Flanagan, Sofie Degerman, Rolf Adolfsson, Rosalba Giugno, Jan Topinka, Xiao-wen Zeng, Anna Oudin

**Affiliations:** 1 Department of Epidemiology and Global Health, Sustainable Health, Umeå University, Umeå, 90187 Sweden; 2 Department of Medical and Translational Biology (MTB), Umeå University, Umeå, Sweden; 3 A.I. Virtanen Institute for Molecular Sciences, University of Eastern Finland, Kuopio, Finland; 4 Department of Laboratory Medicine, Lund University, Lund, Sweden; 5 Department of Medical Biosciences, Umeå University, Umeå, Sweden; 6 Department of Clinical Microbiology, Umeå University, Umeå, Sweden; 7 Department of Clinical Sciences, Umeå University, Umeå, 90187 Sweden; 8 Department of Computer Science, University of Verona, Verona, Italy; 9 Department of Toxicology and Molecular Epidemiology, Institute of Experimental Medicine CAS, Prague, Czech Republic; 10 Department of Preventive Medicine, School of Public Health, Sun Yat-sen University, Guangzhou, China

**Keywords:** Air pollution, Particulate matter with a 2.5 micrometer or less in diameter, Relative leukocyte telomere length, Dementia, Molecular biology, Environmental sciences

## Abstract

Air pollution is increasingly discussed as a risk factor for dementia, but the biological mechanisms are not yet fully understood. Biological markers like telomere length are relevant to study with air pollution, as they are associated with aging and dementia. The study aimed to investigate the relationship between source-specific air pollution exposure and telomere length in a low-level air pollution area, and whether this potential relationship depended on future dementia status. The data originated from the Betula study in Northern Sweden, where 509 participants recruited between 1988 and 1995 were included to investigate the association between annual mean air pollution concentrations at the participants’ residences and relative leukocyte telomere length using a linear regression model. No association was observed between air pollution and telomere length, with regression slope estimates close to zero and p-values > 0.10 (e.g. PM_2.5__total: β = 0.01 (-0.011, 0.025) and BC_total: β = 0.03 (95% CI: -0.046, 0.114). There were indications of a positive association between longer telomere length and higher exposure to air pollution among individuals later diagnosed with dementia (*N* = 74), but these findings were not conclusive (p-values > 0.10) (PM2.5_total: β = 0.03, p-value = 0.12; BC_total: β = 0.11, p-value = 0.17). Although not statistically significant, our findings contribute to the evidence from low-exposure settings, and it is important to report these types of findings for a balanced understanding of potential health effects.

**Supplementary Information:**

The online version contains supplementary material available at 10.1038/s41598-025-19469-7.

## Introduction

Ambient (outdoor) air pollution is a major global health concern^1^. The World Health Organization (WHO) estimates that ambient air pollution causes 3.5 million premature deaths annually and that 99% of the world’s population are currently being exposed to levels above the current air quality guidelines outlined by WHO in 2021^2^. Even at relatively low concentrations, adverse health effects of air pollution thus persist, highlighting its ongoing harm to human health^2^. Investigating air pollution at low exposure levels is crucial because even low levels of exposure can have significant health impacts, especially in vulnerable populations such as children, the elderly, and those with pre-existing conditions. Recent evidence suggests that there may be no safe threshold for certain air pollutants, meaning adverse effects can occur even below current regulatory limits. Recent research furthermore suggests that the association between air pollution and various health oucomes may not be linear, but actually, possibly even steeper at lowe concentrations. Understanding the health risks associated with low-level exposures can inform more protective public health policies and contribute to reducing the overall burden of disease linked to air pollution. This underscores the importance of studying air pollution effects across the full exposure spectrum, not just at high levels.

Extensive evidence from large-scale studies and meta-analyses has demonstrated associations between exposure to ambient particulate air pollution, and the development of dementia^3^. With an ageing population, dementia is now the leading cause of death in some European countries, for example the UK and Belgium^4,5^. Alzheimer’s disease (AD) stands as the primary cause of dementia and is characterized by various pathological features, including the buildup of amyloid-β plaques and neurofibrillary tau tangles, which result from complex interactions among genetic and lifestyle factors^6^. Given the absence of a definitive therapy for AD and other dementia disorders, it is imperative to identify biomarkers that can help predict an individual’s susceptibility to the disease. Notably, air pollution, as a modifiable environmental risk factor, presents an opportunity to address the risk of dementia^6,7^. It is thus furthermore imperative to understand the role of air pollution on such potential biomarkers.

With the ultimate goal of preventing and better treatments for AD and other dementia disorders, a deeper understanding of the molecular-level impact of air pollution and its effects on cellular biomarkers is crucial. Telomeres, the non-coding ends of chromosomes, play a pivotal role in maintaining genomic stability and integrity by safeguarding the coding genome^8^. These protective caps prevent chromosome erosion and fusion, but successively shorten during DNA replication and cell division. Comprised of repeated DNA sequences and specialized proteins, telomeres form a complex structure crucial for cellular health and longevity^9^. Accumulating evidence has linked shorter relative leukocyte telomere length (rLTL) with aging, AD^10^, age-related diseases and mortality^11^. Research indicates that telomeres undergo progressive shortening with age, but various environmental and lifestyle factors can expedite this process. The harmful effects of air pollutants are rooted in inflammation, characterized by oxidative stress and systemic inflammation, raising concerns about their potential impact on cellular integrity and aging as well as overall health. Unravelling the effects of air pollution on telomere length can thus provide insights into the underlying mechanisms through which environmental factors influence human health and disease progression.

According to a systematic review of 12,058 subjects from 2018, exposure to air pollution (long-term, short-term or occupational exposure) may shorten telomere length^12^. In a systematic review from 2019, only four studies were deemed high enough quality for a meta-analysis however, and the meta-estimate was inconclusive^13^.

Health effects of air pollution in general, and inflammatory effects specifically, can be expected to differ depending on the composition of air pollution, both in terms of dominating sources of local air pollutants and the proportion of pollution coming from near sources or from long distance transport. It has for example been suggested that air pollution from sources nearby is more harmful for health than regional-level air pollution^14^. To date, no previous study on air pollution and telomere length has distinguished between different sources of air pollution, or between local or regional air pollution. Previous studies have reported mixed results^15^. Furthermore, research in regions with low air pollution levels and high-resolution pollution models is lacking.

This study aims to address this gap by exploring the association between air pollution and rLTL in a low-level air pollution area while distinghuishing between different sources of local air pollution, and between local and total air pollution (where the latter include air pollution from long-range transport).

## Materials and methods

### Study population

**Fig. 1:**
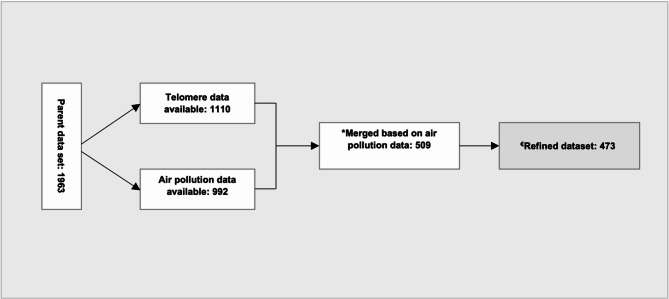
Flow chart illustrating the participant selection process for the study. *Overlap of both datasets. ^€^Based on residualization of telomere against age and gender variables.

The Betula cohort is a longitudinal, population-based study on dementia, memory and ageing which was initiated in 1988 to investigate health and cognitive trajectories in a representative fraction of the adult and elderly population residing in Umeå county, located in the Västerbotten region of Sweden. The comprehensive recruitment process for the Betula study has been extensively detailed in previous publications^16^. In summary, participants in the Betula cohort have undergone examinations up to seven different time waves (T1 to T7), spaced at five-year intervals from 1988 to 2017. Each assessment includes the administration of health-related questionnaires, examinations, and cognitive evaluations. For the purposes of the present study, participants enrolled during the time periods of 1988 to 1990 (test wave 1; T1) and 1993 to 1995 (test wave 2; T2) were selected. In the Betula study, dementia status was assessed at baseline and every five years using DSM-IV criteria, identifying when cognitive symptoms impaired daily functioning. Diagnoses were based on study visit evaluations, supplemented by comprehensive medical records, including neuroimaging and CSF biomarkers when such information was available in medical records. The cohort (*n* = 4,445) was evaluated after each test wave (T1–T5), with diagnoses coordinated by the same senior geropsychiatrist throughout. Predetermined criteria—such as MMSE ≤ 23, cognitive decline, functional impairment, or reported memory loss—triggered extended evaluations^17,18^. We included participants from T1 and T2 of the Betula cohort as the parent sample (labeled as ‘Parent sample’ in Fig. 1) for this study. From this sample, we identified subsets with available data on telomere and air pollution.

### Relative leukocyte telomere length

The relative leukocyte telomere length (rLTL) was measured by quantitative polymerase chain reaction (qPCR) in DNA from whole blood extracted with the Kleargene XL blood DNA extraction kit (LGC Genomics Ltd., UK), drawn during the second test wave (T2) between 1993 and 1995 for a total of 509 included participants. rLTL measurements were conducted in 2014 by the qPCR method for rLTL measurement originally described by Cawthon in 2002^19^. In this study, this method was used with some minor modifications^20^. Each sample was evaluated by Telomere (TEL) and single copy gene hemoglobin subunit beta (HBB, Gene ID:3043) PCR reactions. A TEL/HBB value were calculated using the 2^−ΔCt^ method, in which ΔCt = average Ct_TEL_-average Ct_HBB_. The rLTL value were obtained by dividing the TEL/HBB value of each sample with the TEL/HBB value of a reference cell line (CCRF-CEM) DNA included in all runs. The rLTL values were further normalized for potential plate effects (between-run variability) by subtracting mean-centered plate effects, estimated through a mixed-effects model with age, gender, age × gender interaction and plate as fixed effects, and individuals as a random effect.

### Air pollution exposure assessment

The annual mean concentrations of fine particulate matter (PM_2.5_) and Black Carbon (BC) were computed by the Swedish Meteorological and Hydrological Institute (SMHI)^21^. Local and regional emission inventories served as inputs for the national dispersion modelling system, and SMHI provided the necessary data for the Gaussian dispersion model simulations to calculate annual mean concentrations of PM_2.5_ and BC. The model exhibited high spatial resolution, with concentrations modelled near major roads and close to smokestacks down to 35 m by 35 m. Additionally, the model incorporated emissions from industrial sources and shipping as point sources in its simulations. Emission factors for various types of vehicles were determined based on the Handbook on Emission Factors for Road Traffic, version 3.1^22^. Non-exhaust emissions, which encompass road wear and some contributions from brake wear and tire emissions, were also quantified. To estimate emissions from residential wood combustion, data from inventories of individual stoves and boilers, along with information gathered from chimney sweepers and interviews regarding wood burning habits, were utilized^23^. The assessment of long-range transport air pollution relied on data from rural background monitoring stations. This involved calculating the difference between the total concentrations measured at these monitoring stations and the modelled local particle concentrations at the same locations. The total PM2.5 concentrations used in this study were based on dispersion modeling previously validated against measured concentrations in multiple Swedish cities, showing reasonably good agreement with an R² of 0.65 (21), which supports the reliability of the exposure estimates used.

Finally, the resulting concentrations of air pollution were linked to each participant’s residential address at sampling, and the modelled value of year 1990 was used as marker for long-term exposure for the participants. Although the exposure assessment was conducted in the early 1990s, the levels in the study area were, and continue to be, low by both national and international standards. Importantly, we examined within-city contrasts, which remain relevant today despite reductions in overall pollution levels. As we were able to model source-specific concentrations of locally emitted PM2.5 and BC from vehicle exhaust and wood smoke, sources that are still dominant in the area, our findings are likely more generalizable to current urban environments than studies relying on total PM2.5 alone. The inclusion of BC, a key pollutant linked to combustion and climate impacts, further strengthens the relevance of our results for contemporary public health policy.

The six exposure variables included total concentrations of PM_2.5_ and BC (PM_2.5__total, BC_total) as well as source-specific concentrations of PM_2.5_ (PM_2.5_ _exhaust, PM_2.5_ _woodburning) and BC (BC_exhaust, BC_woodburning). The study area, Umeå municipality, is located in Northern Sweden, and has moderate levels of PM_2.5_, with a total concentration of 9.81 µg/m^3^ in the present study (Table 1). This is below the former WHO recommended air quality guideline from 2005 of 10 µg/m^3^, but over the revised WHO (in 2021) air quality guideline of 5 µg/m^3^.

### Statistical analysis

The calculation of blood lymphocyte proportion involved dividing the lymphocyte count by the total count of white blood cells, which encompasses neutrophils, eosinophils, basophils, lymphocytes, and monocytes. To mitigate the influence of age and gender-related variations, relative leukocyte telomere length (rLTL) was residualized against the participant’s age at the time of rLTL measurement and their gender, using a linear regression model. This yielded a residualized telomere length, denoted as rLTL, which was then utilized to investigate the relationship between air pollution and telomere length. We considered age, gender (female, male), lymphocyte proportion, education level (compulsory, high school, university), and smoking status (smoker or former smoker, non-smoker) as a potential confounding factors to be included in this study. Air pollution exposure, rLTL as well as all potential confounders were assessed at test wave T2, whereas dementia was assessed with follow-up to February 2022.

Linear regression analysis was employed to assess the impact of both total and source-specific particle concentrations on rLTL. Model 1 was an unadjusted model. Model 2 incorporated adjustments for lymphocyte proportion, age and gender. Model 3 (the main model) additionally accounted for individual-level baseline potential confounders, including education level and smoking status. We created Q-Q plots to evaluate the assumptions of linearity and normal distribution in linear regression (Figure. S1, S2, and S3) in the Supplementary material.

Furthermore, subgroup analysis based on future dementia diagnosis (Dementia, No dementia) was carried out to evaluate whether the association between air pollution and rLTL differed depending on dementia status. Here, a linear regression model was used and a multiplicative interaction term was included in the main model between each of pollutants and dementia status to explore potential effect modification. All statistical analyses were performed using R version 3.4.8.

The study was approved by the Ethical Review Board in Umeå with Dnr: 2022-04608-01, and written informed consent was obtained from all Betula participants. The data for the present study were accessed for research purposes on March 15, 2023. The researchers analyzing the data did not have access to information that could identify individual participants. All methods were conducted in accordance with relevant guidelines and regulations.

## Results

In the Betula study sample, 1110 individuals initially had their rLTL measured during T2. Data was available for 509 individuals after matching with air pollution exposure data. Furthermore, residualization of rLTL against age and gender varibles resulting in a refined dataset consisting of 473 participants for initial analysis. Detailed characteristics of these participants can be found in Table 1.

**Table 1:** Characteristics of the participants.

Variables	Number of observations	Values^a^
Age	473	60.8 (14.7)
Gender		
Female	251	53.1
Male	222	46.9
Education level		
Compulsory	158	47.4
High school	223	33.6
University	90	19.0
Smoking status		
Former smoker or Smoker	224	47.6
Non-smoker	247	52.4
^b^Lymphocyte proportion	461	0.30 (0.08)
^c^PM_2.5_ _total	473	9.81 (0.66)
^d^ PM_2.5_ _exhaust	473	0.24 (0.25)
^e^PM_2.5_ _wood	473	1.24 (0.31)
^f^Black C_total	473	0.56 (0.15)
^g^BC_exhaust	473	0.11 (0.12)
^h^BC_wood	473	0.16 (0.04)
Dementia status		
Dementia	75	15.9
No dementia	398	84.1

^a^Values are mean (standard deviation) for continuous variables, percentage for categorical variables.

^b^Lymphocyte proportion was calculated as lymphocyte count divided by the sum of all white blood cells count (sum of neutrophils, eosinophils, basophils, lymphocytes, and monocytes).

^c^Total PM_2.5_ (particulate matter with an aerodynamic diameter of ≤ 2.5 micrometers, “fine” particulate matter) concentration.

^d^Particle emission from vehicles on the road.

^e^Particle emission from wood burning.

^f^Total black carbon concentration.

^g^Black carbon emission from vehicles on the road.

^h^Black carbon emission from wood burning.

The age of the participants ranged from 40 to 85 years (mean: 60.8 years, standard deviation (SD): 14.7). Among this group, 53.1% were female, 47.6% were smokers, and 19% had completed a university degree. The annual mean concentrations (SD) of total PM_2.5_ and BC were 9.81 (0.66) µg/m^3^ and 0.56 (0.15) µg/m^3^, respectively for 1990 (used as marker for long-term exposure in the study). During the follow-up period up to 2016, 15.9% developed dementia up to 2022, and the proportion of females in the dementia group was higher (53%) than the proportion of males (47%). The mean rLTL value exhibited variation depending upon whether participants would later be diagnosed with dementia or not. Shorter mean rLTL values (-0.0072) were observed among study participants who later were diagnosed with dementia than those who were not (0.00135), however, the difference was not statistically significant (p-value = 0.61).

We also compared the participants included in the study with those excluded due to missing air pollution and telomere data to assess potential selection bias. The two groups were generally similar; however, excluded participants were, on average, younger, had a slightly higher proportion of females, and were more likely to have a university education (Supplementary Table S1).

The linear associations between air pollution and residualized leukocyte telomere length (rLTL), are presented in Table 2.

**Table 2:** Associations between air pollution and relative leukocyte telomere length (β, 95% CI).

Measures	Model 1 (*N* = 473)	Model 2 (*N* = 462^*^/461^**^)	Model 3 (*N* = 460^*^/459^**^)
Air pollution exposure (µg/m^3^)	β (95% CI)	β (95% CI)	β (95% CI)
PM_2.5__total	0.01 (− 0.009, 0.026)	0.01 (− 0.011, 0.024)	0.01 (− 0.011, 0.024)
PM_2.5__exhaust	0.03 (− 0.019, 0.074)	0.03 (− 0.023, 0.072)	0.02 (− 0.023, 0.072)
PM_2.5__woodburning	0.01 (− 0.025, 0.049)	0.01 (− 0.33, 0.042)	0.01 (− 0.033, 0.044)
BC_total	0.04 (− 0.036, 0.119)	0.04 (− 0.042, 0.116)	0.03 (− 0.046, 0.114)
BC_exhaust	0.06 (− 0.039, 0.155)	0.05 (− 0.046, 0.152)	0.05 (− 0.019, 0.042)
BC_woodburning	0.13 (− 0.194, 0.452)	0.11 (− 0.165, 0.432)	0.11 (− 0.165, 0.428)

Model 1: Unadjusted; Model 2: Model 1 + Age, gender and lymphocyte proportion; Model_3: Model 2 + smoking and education. * No of observation in models with PM_2.5_ exposure variable. ** No of observation in models with BC exposure variable.

Notably, small but imprecise tendencies for rLTL to increase with air pollution exposure were observed, especially for the BC elements. For example, higher concentrations of BC were associated with longer rLTL: a 1 µg/m^3^ increase in total BC was associated with a unit increase of 0.03 (95% CI: − 0.046, 0.114) in rLTL in Model 3 (Table 2). The corresponding estimates for PM_2.5__exhaust, PM_2.5__woodburning, BC_exhaust and BC_woodburning were: 0.02 (95% CI: − 0.023, 0.072), 0.01 (95% CI: − 0.033, 0.044), 0.05 (95% CI: − 0.019, 0.042), and 0.11 (95% CI: − 0.165, 0.428), respectively.

The estimates from the subgroup analysis based on dementia status are reported in Table 3.

**Table 3:** Associations between air pollution and relative leukocyte telomere length stratified by dementia.

Air pollution exposure (µg/m^3^)	Dementia (74)	No dementia (385)	*p*-value^c^
	β^a^ (*p*-value)^b^	β^a^ (*p*-value)^b^	
PM_2.5__total	0.03 (0.12)	− 0.001 (0.92)	0.14
PM_2.5__exhaust	0.06 (0.14)	0.004 (0.89)	0.24
PM_2.5__woodburning	0.034 (0.48)	0.0004 (0.99)	0.47
BC_total	0.11 (0.17)	0.003 (0.96)	0.21
BC_exhaust	0.12 (0.22)	0.01 (0.88)	0.28
BC_woodburning	0.41 (0.35)	0.073 (0.70)	0.59

^a^Residualized model coefficients (Model 3).

^a^Adjusted for age, gender, lymphocyte proportion, smoking status and education level.

^c^P-values for interaction between pollutants and dementia status).

Associations between air pollution exposure and rLTL appeared to be present mainly in study participants who later developed dementia, but all p-values are larger than 0.10, so the results are not conclusive. The interaction terms for effect modification by dementia status were furthermore not statistically significant.

Visual inspection of Q-Q plots for residuals shows a nearly normal distribution, as points closely follow the theoretical quantile line, as demonstrated in Supplementary material Figure S1 to S3.

## Discussion

In this study, associations between air pollution and telomere length were not evident. Although not statistically significant, there were however some tendencies for telomere length to increase with exposure to ambient particles among study participants who were later diagnosed with dementia. Overall, the results diverge from our initial hypothesis however, which anticipated air pollution to be associated with a *decreased* telomere length. The statistical precision of the estimates were low. Consequently, these findings should be interpreted cautiously and corroborated in other studies. If we speculate however, the results raises questions about susceptibility to air pollution and about the state of the inflammatory response in people who develop dementia. The idea that susceptibility to air pollution may differ between different groups in the population is not new; we have for example previously observed that associations between particulate air pollution and dementia were mainly present among study participants carrying the *APOE* ɛ4 allele and for those with low performance on odor identification ability in the same cohort^24^.

Previous research on air pollution and telomere length have yielded somewhat conflicting findings although long-term exposure appears to be associated with shorter telomere length in several studies^12,13^. In a study using cross-sectional data on 471,808 UK Biobank individuals, telomere length decreased with increases in long-term concentrations of air pollution^25^. Another large study conducted on UK Biobank participants, however, found no significant association between long-term exposure to several pollutants and telomere length,^26^. Discrepancies may thus occur in the same cohort, the main difference between the two studies seem to be the air pollution modelling. In alignment with our findings, a study involving school children in East London demonstrated an increase in telomere length with rising long-term concentrations of air pollution^27^. Interestingly, genetic ancestry seemed to influence the associations in that study, with stronger associations being observed in black children. In the present study, associations seemed stronger in people who later developed dementia, which is also linked to genotype. In a study from Shanghai, China, on patients with diabetes mellitus, no associations between short-term levels of air pollution and telomere length was observed^28^. In a heterogenous cohort of critically ill patients it was observed that long-term exposure to air pollution was positively associated with telomere length^29^. The authors speculate that an interaction of air pollutant exposure and acute inflammation may activate telomerase in these patients^29^. These finding raises questions about whether the inflammatory response in these individuals differs from the general population.

The mixed findings in previous studies underscore the complexity of the relationship between air pollution and telomere length, suggesting that multiple factors may contribute to the observed results. The levels and composition of air pollution may evidently partly explain some of the discrepancies in findings. The role of short-term versus long-term exposure to air pollution is potentially influencing the results as well. In the present study, we used annual mean concentrations of air pollution as a marker for long-term exposure to air pollution. We did not have access to short-term measurements. It has been proposed that the acute, toxic effects of inflammation following short-term air pollution exposure, such as one’s average daily exposure, may lead to telomere lengthening by the activation of the enzyme telomerase^30^. In the present study we find it unlikely that short-term exposure would influence the results however, since we have previously seen that the correlation between short-term levels and long-term exposure is very low in the Betula study^31^.

Another potential explanation for the disparity in results in previous studies is the realm of proinflammatory effects of air pollution and differential distribution of leukocytes in response to inflammation. Telomere measurement is conducted as a weighted average of different leukocytes subtypes and it is important to note that proportion of neutrophils in blood circulation is higher than other leukocytes subtypes and they have longer telomere length than lymphocytes^32,33^. Notably, acute inflammation is characterised by predominant accumulataion of neutrophils rather than lymphocytes, thus, it is plausible to presume that inflammation as a result of air pollution exposure may cause longer telomere length due differential clonal expansion of leukocytes^30^. In the present study however, we controlled for lymphocyte proportion in an attempt to control for such differential expasions. Another mechanism by which air pollution may increase the telomere length is based on inflammation-related oxidtative stress. Although, oxidative stress has been suggested to decrease telomere length but contrarily it may also cause an increase in telomere length by pairing up four guanine residues in the DNA strand^34^. This enhances the accessibility of telomerase enzyme which eventually causes an increase in telomere length.

There are several strengths and limitations of the present study that should be acknowledged. One of the key strengths is the estimation of air pollution concentrations using a high-resolution dispersion model. These estimates were further refined by accounting for meteorological conditions, traffic volume (including vehicle types and speed), street width and neighbouring building heights. Additionally, this study is among the first, to our knowledge, to examine the effects of source-specific particles on telomere length. The validation of the source specific concentrations were less rigorous than the total concentrations however, so these estimates should be interpreted with more caution. For wood-burning, rigorous validation has been done in small villages outside the study area, with very high correlations. Other strengths are the detailed dementia diagnosis and the long follow-up time within the Betula study^18^. There are also several limitations to consider. Firstly, telomere length measurements with qPCR are known to suffer from measurement imprecision, although qPCR is still the preferred method in epidemiological studies due to its cost-effectiveness^35^. There is furthermore a risk of bias or lack of precision due to self-reported covariate information. There may also be residual confounding. We adjusted for level of education, which previous research has confirmed to increase with telomere length^36^, but residual confounding due to socio-economy or lifestyle factors may have biased the estimates. Furthermore, chronic conditions like cardiovascular diseases and COPD has been shown to influence telomere length and are correlated with air pollution exposure, at least in settings with higher pollution levels^37,38^. It is possible that such associations may have confounded the results in the present study, and future studies should consider including more information about comorbidities^39^. The generalizability of our study is furthermore limited as it focuses on a specific geographic location with relatively low air pollution levels compared to other regions worldwide. However, the source-specific estimates should be reasonably generalizable to other similar areas in the world. Additionally, our study relies on cross-sectional observational data, which makes it challenging to establish causal inferences. However, follow-up for dementia incidence was conducted longitudinally. Moreover, our previous studies in the Betula study shows high correlation between long-term exposure to air pollution and the annual mean (which was used in the present study). We, furthermore, acknowledge the possibility of selection bias, as some participants with short residual leukocyte telomere length (rLTL), which is, again, associated with aging, age-related diseases and mortality, may have died before study enrolment. This could potentially lead to an underestimation of the effect estimates between air pollution and rLTL. Furthermore, excluding participants because of missing air pollution and telomere data may lead to selection bias. Although the excluded participants were generally similar to those included, some differences exist that could still introduce bias.

Given that the levels of air pollution was low and the study size was limited compared to for example the studies in the UK biobank^25,26^, the statistical power to detect associations could furthermore be too low to detect associations. The particle levels were well above the WHO guidelines for PM2.5 from 2021 however, and in previous studies in Betula we have been able to detect associations between air source-specific pollutants and dementia^24,40^. Although the sample size in our study limits the statistical power, particularly for the dementia analysis, the observed associations, regardless of statistical significance, offer valuable insight into potential links between air pollution and health. It is important to emphasize that effect estimates can still inform the scientific and policy discourse, especially when aligned with existing literature, or as provider of new hypotheses. These limitations underscore the need for replication in larger cohorts. Nonetheless, our sub-analyses based on comorbidities provide important exploratory findings. Identifying potentially more vulnerable subgroups can guide future research and help prioritize public health interventions in urban planning and policy. Another potential weakness is that we used the 1990 annual mean exposure as a proxy although the rLTL measurements were done in samples collected 1993–1995. Spatial contrasts in air pollution within the study area are known to be highly stable over short time periods. Additionally, we have examined potential bias from residential mobility in longitudinal cohorts within this area^41^. This experience gives us confidence that the relative differences in exposure between participants remain consistent, supporting the validity of using 1990 data to represent individual exposure at the time of rLTL measurement in 1993–1995.

## Conclusion

In summary, we did not observe any clear assocition between air pollution and leukocyte telomere length. In people who later developed dementia, there were tendencies for air pollution to be associated with an increase in leukocyte telomere length. Despite established health risks associated with air pollution, its specific impact on telomere length is a complex and evolving field. Conflicting study results highlight the need for further investigation to unravel the intricacies and discrepancies, shedding light on underlying mechanisms. Future research should focus on exploring the associations between air pollution exposure and telomere length, with attention to susceptible groups and the mechanisms involved. Future studies should also consider incorporating a broader range of aging biomarkers, such as epigenetic clocks, mitochondrial DNA damage, and proteomic or metabolomic signatures, to more comprehensively capture the complex biological pathways through which air pollution influences the aging process.

## Supplementary Information

Below is the link to the electronic supplementary material.

Supplementary Material 1

Supplementary Material 2

Supplementary Material 3

Supplementary Material 4

## Data Availability

The data that support this study’s findings are accessible through Region Västerbotten, but there are restrictions on their availability. These data were utilized under license for the current study and are not publicly available. However, the authors (corresponding author) can provide the data upon reasonable request and with permission from Region Västerbotten.

## Abbreviations

AD: Alzheimer’s disease
BC: Black carbon
HBB: Hemoglobin subunit beta
PM2.5: Particulate matter with a diameter of 2.5 micrometer or less
qPCR: Quantitative polymerase chain reaction
rLTL: Relative leukocyte telomere length
SD: Standard deviation
SMHI: Swedish Meteorological and Hydrological Institute (SMHI)
TEL: Telomere
WHO: World health organization
