# Supplementary material for: Associations between air pollution and relative leukocyte telomere length among northern Swedish adults based on findings from the Betula study
Source: Sci Rep. 2025 Sep 23;15:32660. doi: 10.1038/s41598-025-19469-7 (PMC12457613; doi:10.1038/s41598-025-19469-7)
Supplement: Supplementary file 1 — Supplementary Material 1 [file 41598_2025_19469_MOESM1_ESM.docx]

**Appendix**

|  | | |
| --- | --- | --- |
| Variables | Number of observations | Values^a^ |
| Age | 601 | 59.7 (14) |
| Gender |  |  |
| Female | 335 | 55.7 |
| Male | 266 | 44.3 |
| Education level |  |  |
| Compulsory | 296 | 50.0 |
| High school | 123 | 21.0 |
| University | 173 | 29.0 |
| Smoking status |  |  |
| Former smoker or Smoker | 276 | 46.0 |
| Non-smoker | 325 | 54.0 |
| ^b^Lymphocyte proportion | 586 | 0.30 (0.08) |
| Dementia status  Dementia  No dementia | 110  491 | 18.3  81.7 |
| **Table S1.** Characteristics of the participants. ^a^ Values are mean (standard deviation) for continuous variables, percentage for categorical variables. ^b^ Lymphocyte proportion was calculated as lymphocyte count divided by the sum of all white blood cells count (sum of neutrophils, eosinophils, basophils, lymphocytes, and monocytes) | | |

**
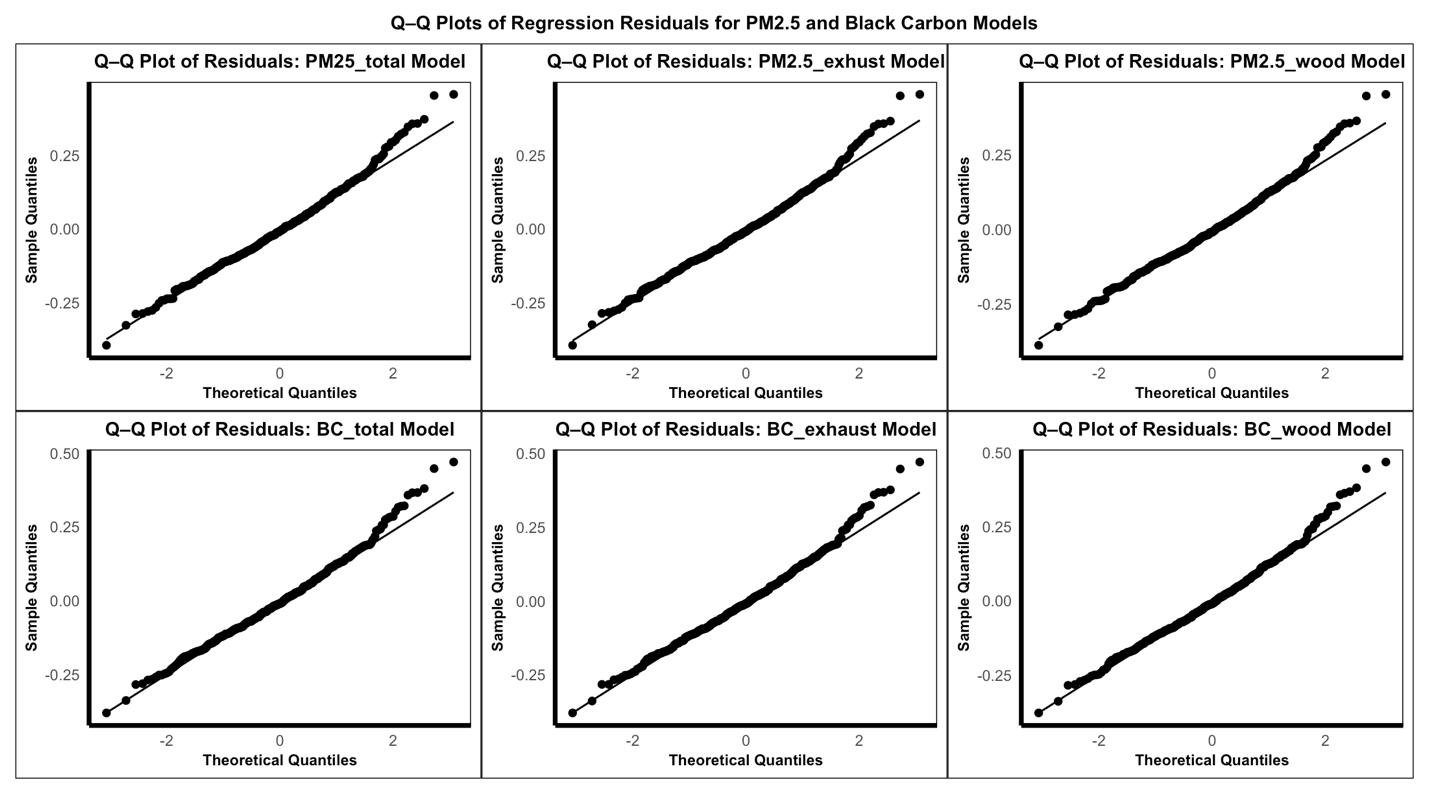
**

Figure S1. Q-Q plots of the residuals from the linear regression model used to assess the normality assumption, illustrating the association between air pollutants and telomere length.


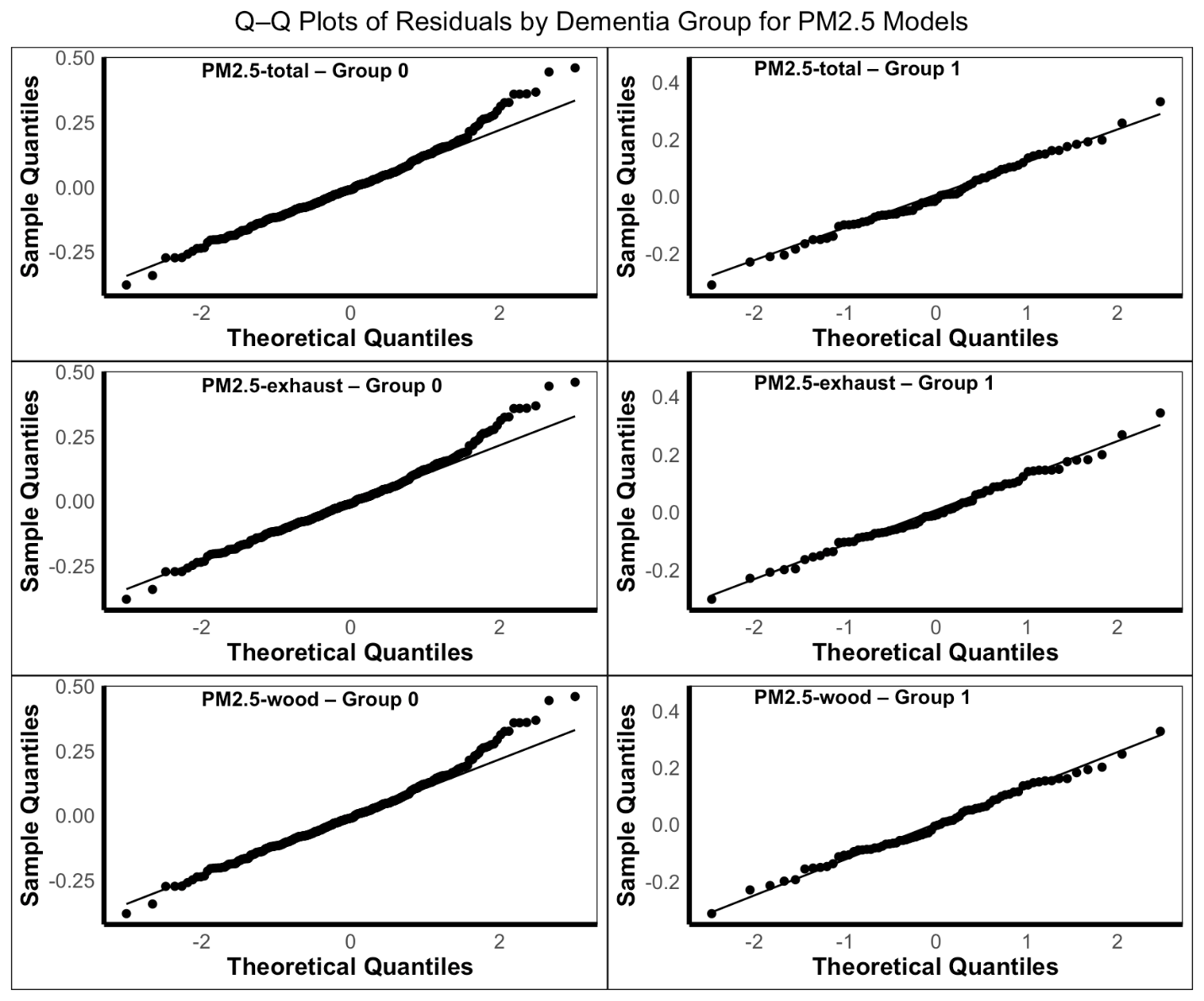


Figure S2. Q-Q plots of the residuals from the linear regression model used to assess the normality assumption, illustrating the relationship between PM_2.5_ and telomere length based on dementia status; Group 0 = No dementia, Group 1 = Dementia.


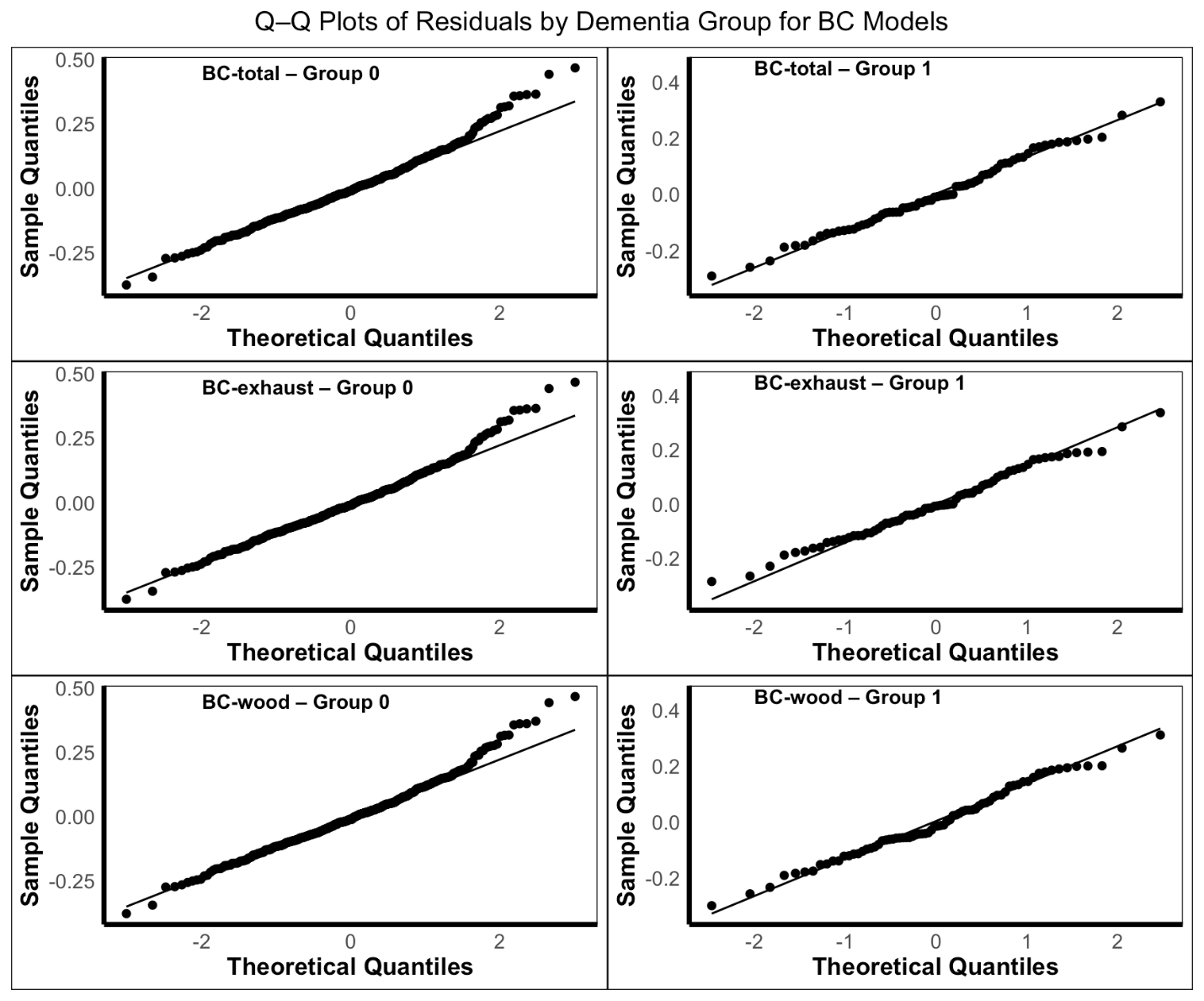


Figure S3. Q-Q plots of the residuals from the linear regression model used to assess the normality assumption, illustrating the relationship between BC and telomere length based on dementia status; Group 0 = No dementia, Group 1 = Dementia.
