# Supplementary figures and images for: Associations between air pollution and relative leukocyte telomere length among northern Swedish adults based on findings from the Betula study
Source: Sci Rep. 2025 Sep 23;15:32660. doi: 10.1038/s41598-025-19469-7 (PMC12457613; doi:10.1038/s41598-025-19469-7)

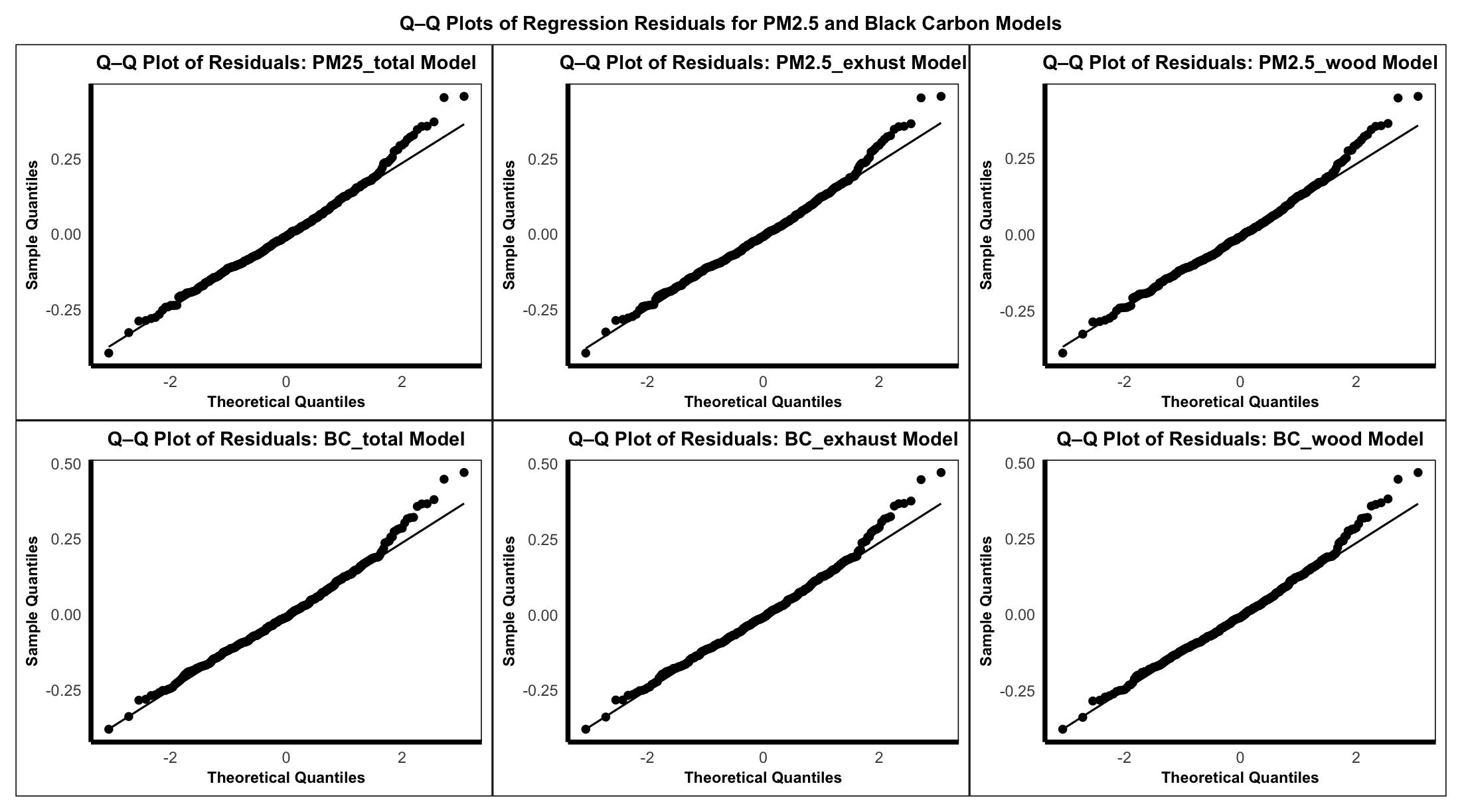

Supplement: Supplementary file 2 — Supplementary Material 2 [file 41598_2025_19469_MOESM2_ESM.png]

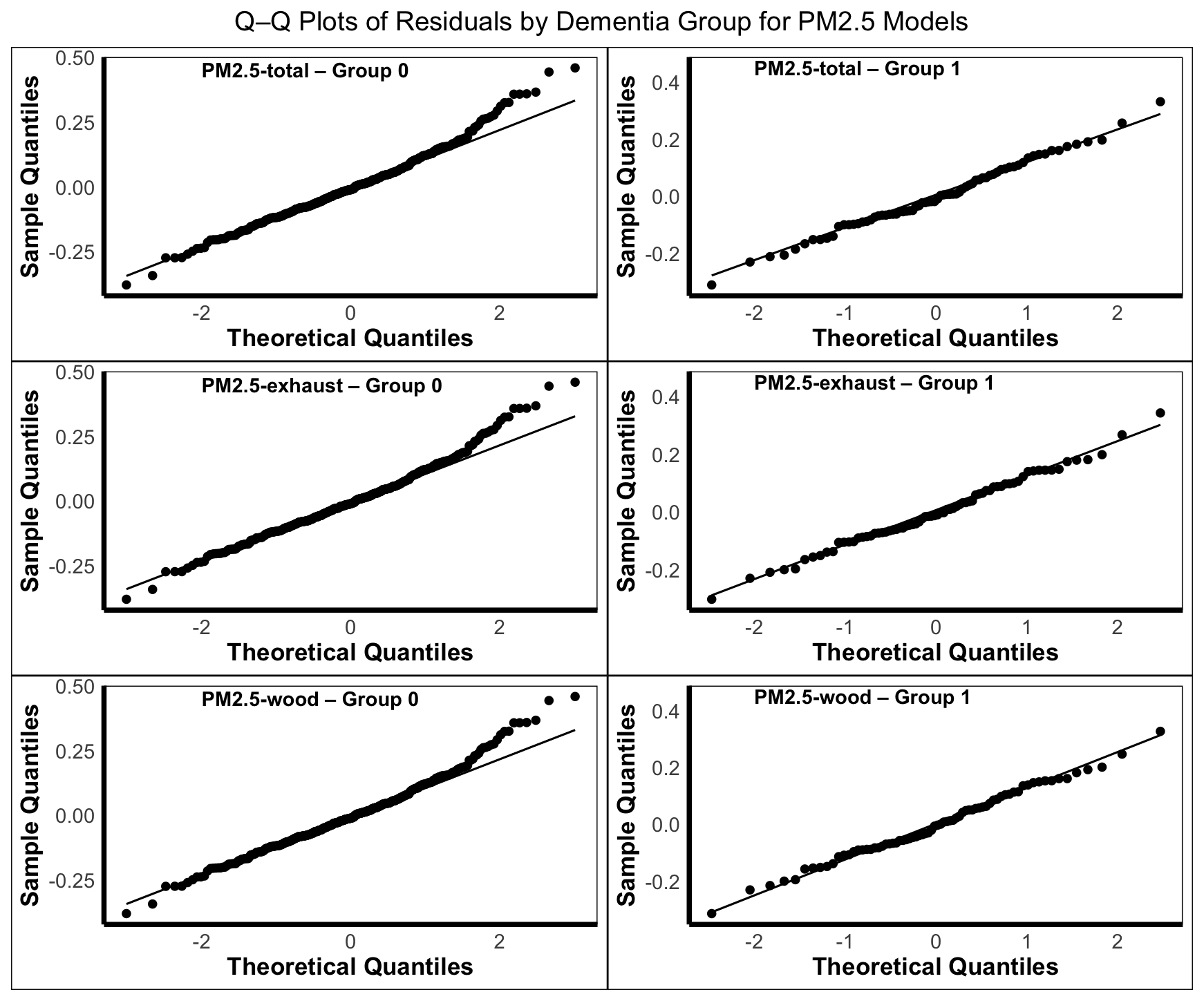

Supplement: Supplementary file 3 — Supplementary Material 3 [file 41598_2025_19469_MOESM3_ESM.png]

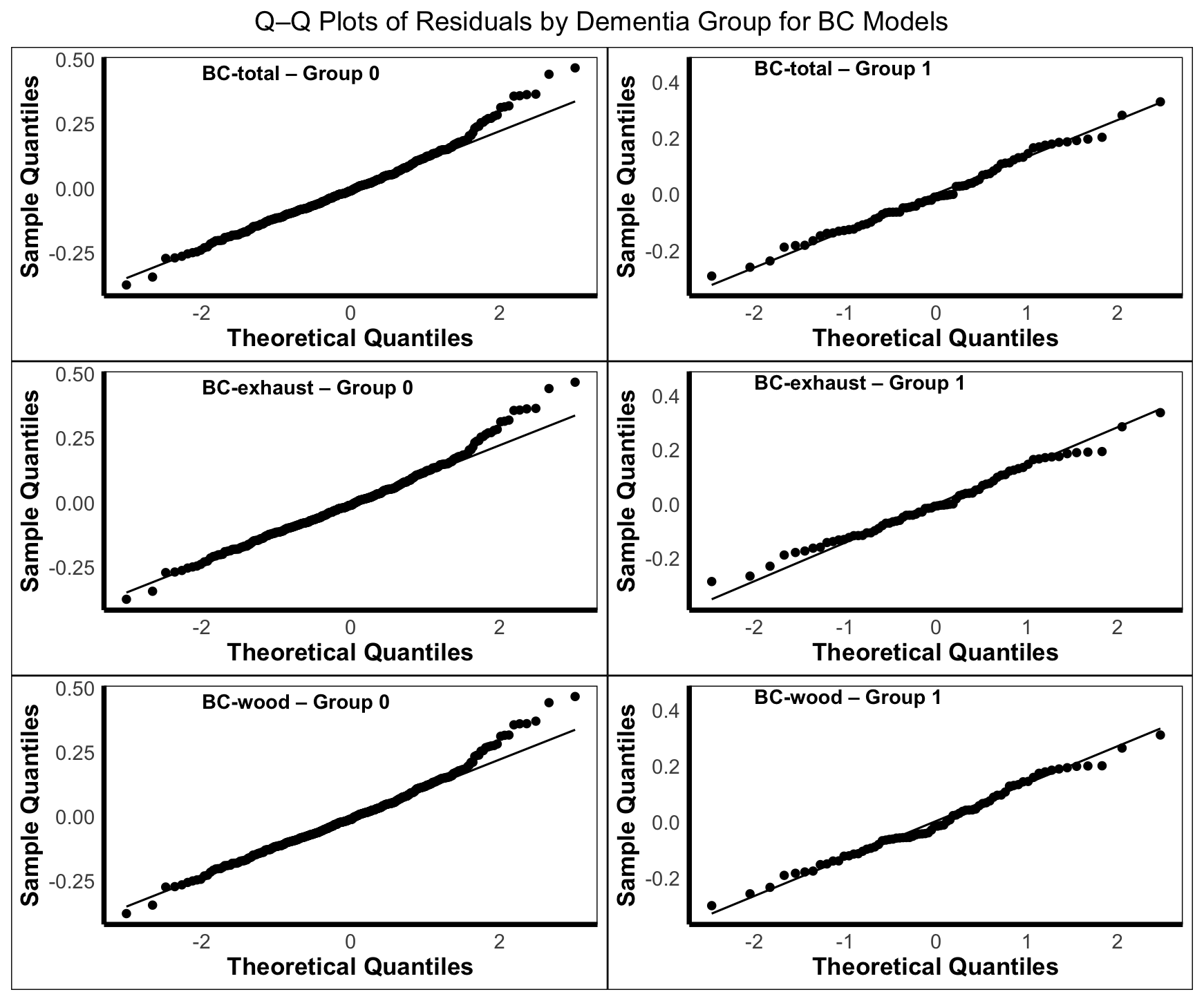

Supplement: Supplementary file 4 — Supplementary Material 4 [file 41598_2025_19469_MOESM4_ESM.png]
